# Adenosine A2A and A2B Receptor Substantially Attenuate Ischemia/Reperfusion Injury in Septic rat Hearts

**DOI:** 10.1007/s10557-016-6693-y

**Published:** 2016-10-18

**Authors:** Hendrik Busse, Diane Bitzinger, Klaus Höcherl, Timo Seyfried, Michael Gruber, Bernhard M. Graf, York A. Zausig

**Affiliations:** 1 Department of Anesthesiology, University hospital of Regensburg, Franz-Josef-Strauss-Allee 11, 93053 Regensburg, Germany; 2 Institute of Experimental and Clinical Pharmacology and Toxicology, Friedrich-Alexander-Universität Erlangen-Nürnberg (FAU), Erlangen, Germany

**Keywords:** Septic heart, Adenosine receptor, Ischemia/reperfusion injury, AdorA2a, AdorA2b

## Abstract

**Introduction:**

Mechanical and morphological ischemia and reperfusion (I/R) injury is reduced in septic hearts. The mechanism behind this “cardioprotection” is less well understood. As adenosine receptors play a major role for cardioprotection in non-septic hearts, we investigated the influence of adenosine receptors in a model of I/R in septic hearts.

**Methods:**

SHAM operation or cecal ligation and puncture (CLP) was performed in adult male Wistar rats (*n* = 60). After 24 h of incubation, hearts were isolated and randomly assigned to a group with or without adenosine receptor (Ador) antagonists (SCH 58261 and MRS 1706) administered before reperfusion. Ischemia and reperfusion lasted for 40 min each. Cardiac function of the heart was determined by measuring left ventricular pressure (LVP).

**Results:**

Before I/R, CLP hearts showed a significant mechanical left ventricular impairment (CLP: 63 ± 5 mmHg vs. SHAM: 104 ± 6 mmHg. After I/R, left ventricular function was significantly reduced in SHAM (24 ± 32 mmHg), but not in CLP hearts (65 ± 13 mmHg). mRNA expression for the AdorA2a and AdorA2b was significantly increased in CLP, but not in SHAM hearts. LVP of CLP hearts deteriorated when AdorA2a and AdorA2b were blocked.

**Conclusions:**

The morphological and functional I/R injury in septic animals is less pronounced compared to non-septic animals. By a combined blockade of AdorA2a and AdorA2b this “cardioprotective” effect is nearly abolished in septic hearts. This is the first study showing, that AdorA2a and AdorA2b may play an important role for a reduced functional I/R injury in the septic heart.

## Introduction

The new sepsis-3 definition describes sepsis as a life-threatening organ dysfunction caused by a dysregulated host response to infection [1]. Pro- and anti-inflammatory responses interact with hormonal, metabolic, neuronal and in particular cardiovascular pathways. Therefore, a generalized micro- and macrovascular dysfunction is found in sepsis [2–4]. This may result in circulatory and cellular/metabolic abnormalities, which may cause septic shock. Even the heart is affected showing myocardial depression and alteration of coronary flow [3]. As sepsis goes along with volatile hemodynamics and a highly demanded heart function, an insufficient coronary blood supply to the myocardium might be plausible. This would also explain why increased cardiac biomarkers (eg cardiac troponin I and T) are frequently found in septic patients. However, there is general agreement that a global ischemia is not the reason for cardiac dysfunction [3]. In fact, the underlying mechanism behind the cardiac dysfunction in sepsis, the so-called septic cardiomyopathy, might rather be multifactorial and myocardial depression factors (cytokines, prostanoids, and nitric oxide, among others) and a diversity of activated cascades (e.g. endothelial activation, induction of the coagulatory system) play prominent roles in this context. In contrast, the changes in coronary function might be interpreted as an adaption of the heart to the elevated requirements in sepsis; and might be more an advantage than a barrier for the flow of coronary blood supply of the heart. [3–5] In this regard, McDonough et al. showed that ischemia and reperfusion injury is reduced in septic hearts [6], because 50 min of ischemia did not affect left ventricular pressure compared to preischemic values. These results were present at various times of ischemic episodes in different kind of models [6–8]. However, the mechanism behind this “cardioprotection” is less well understood [6].

In healthy hearts, there is substantial evidence that the purine nucleoside adenosine plays a major role in ischemia-reperfusion (I/R) injury [9–11]. Administration of adenosine prior to ischemia or at onset of reperfusion protects the heart from damage. This cardioprotective effect is triggered via the activation of membrane adenosine receptors. These receptors are G-protein-coupled receptors, and of the existing four subtypes (adenosine receptor: Ador; AdorA1, AdorA2a, AdorA2b and AdorA3) all are expressed in the heart. [12] Each of them shows cardioprotective effects, while protection seems to be most beneficial via the activation of AdorA1 and AdorA3 before ischemia, and the stimulation of AdorA2a, AdorA2b during reperfusion [9, 10, 12, 13]. Administration of selective AdorA1 receptor agonists like AMP 579 or GR79236 before ischemia improved postischemic flow and cardiac contractility, and reduced infarct size in both isolated hearts and intact animals in multiple species like rat and canine [11, 13, 14]. Stimulation of AdorA2a at the onset of reperfusion leads to markedly decrease in I/R injury. For example, Norton et al., showed a dose dependent effect of a selective AdorA2a agonist CGS-21680 leading to a significant reduction in histologically determined infarct size [13]. The AdorA2b as well as the AdorA3 type are both so called low-affinity receptors. Therefore, in this case large doses of adenosine or analogues are necessary for a cardioprotective effect. This might be the reason why there are conflicting results showing that the administration of agonists (NECA and BAY 60–6583) or even antagonists (DPCPX and BG 9928) are capable to reduce infarct size in different models [11, 15, 16]. Little is known about the cardioprotective effect of AdorA3. There are many problems like the expression in the heart is very low, a lack of validation, missing specific antibodies and significant species differences in AdorA3 structure and pharmacology [11]. A recent study by Ge et al., showed AdorA3 activation during reperfusion reduces infarct size [17]. In conclusion, adenosine receptors play an important role in I/R injury in healthy hearts. The effect of adenosine receptors in the context of sepsis with special focus on the heart and I/R injury has not been studied so far. Therefore, the aim of this study was to test (1) the hypothesis that septic hearts are “cardioprotected” against I/R injury, and (2) if so, whether adenosine receptors are involved.

## Materials and Methods

### Animals

Approval from the Institutional Animal Care Committee of the University of Regensburg, Germany (54–2532.1-24/09) was obtained before initiation of this study. Animal experiments have been conducted in accordance to the German laws regulating animal care, the European Communities Council Directive and institutional guidelines. Experiments were conducted using adult male Wistar rats (weighing 276 ± 55 g) purchased from Charles River (Charles River, Sulzfeld, Germany).

### Cecal Ligation and Puncture (CLP)

After intraperitoneal injection of 100 mg • kg^−1^ ketamine and 5 mg • kg^−1^ xylazine hydrochloride, SHAM operation (only laparotomy) was carried out or cecal ligation and puncture (CLP) was performed. CLP was performed as described in detail before [18, 19].

### Isolated Heart Preparations

After 24 h of incubation, hearts were isolated and the aorta was rapidly cannulated. During preparation hearts were continuously retrogradely perfused with a cold (6.2+/−0.2° Celsius), oxygenated, modified Krebs-Ringer solution and transferred to a Langendorff apparatus (Hugo Sachs Electronic KG, March-Hugstetten, Germany) [18]. The modified Krebs-Ringer’s salt solution, was filtered in-line (5 μm pore size filter disk, Sigma-Aldrich®, Munich, Germany) and had the following composition: Na^+^ 140 mM; K^+^ 4.5 mM; Mg^2+^ 1.2 mM; Ca^2+^ 2.5 mM; Cl^−^ 134 mM; HCO_3_^−^ 15.5 mM; H_2_PO_4_^−^ 1.2 mM; EDTA 0.05 mM; glucose 11.5 mM; pyruvate 2 mM; mannitol 10 mM; and insulin 5 units per liter. Krebs-Ringer solution was equilibrated with 95 % oxygen and 5 % carbon dioxide. A fluid-filled balloon was introduced into the left ventricle. LVEDP was kept at 0 mmHg during the stabilization period. Hearts were perfused with a constant flow of 10 ml/min and were paced at 350 beats/min in the ischemia and reperfusion period. Left ventricular pressure (LVP), heartrate and coronary flow and pressure were continuously measured, recorded digitally and displayed on a screen. Throughout the experiment all hearts were kept in a glass cage (from Hugo Sachs, Germany) at constant temperature (36.2+/−0.6 °C).

To determine maximal coronary flow reserve, adenosine was injected (0.2 mL of a 200 μM stock solution) directly into the aortic root cannula during the initial control period 15 min before and after the experiments. Post-I/R effluent was collected and lactate (photometric), CK-MB and troponin I (both luminescence oxygen channeling assay) were determined.

### Protocol 1

**Fig. 1:**
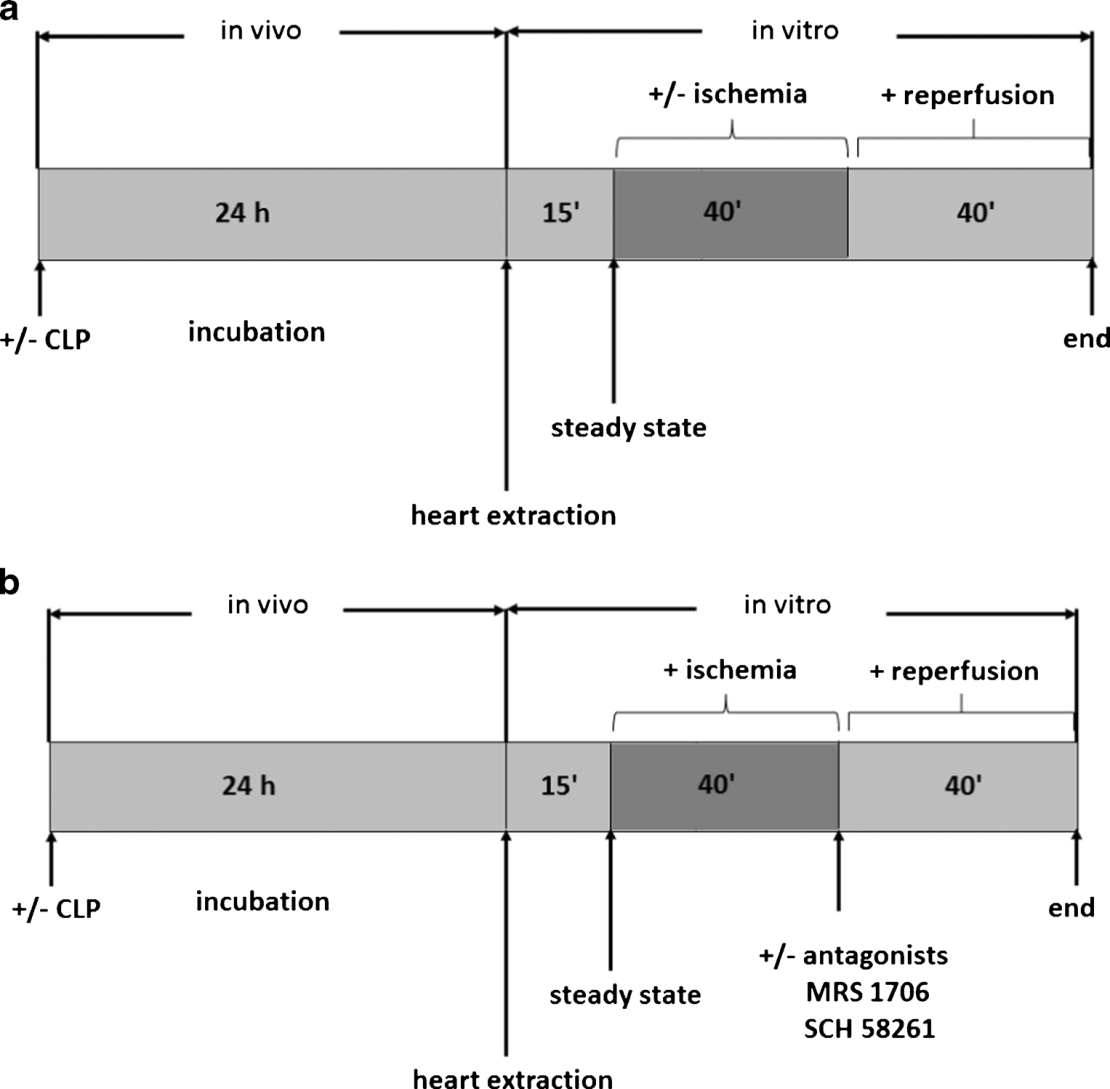
(**a**) Protocol 1; (**b**) protocol 2; CLP = cecal ligation and puncture

After a stabilization period of 15 min, hearts were randomly assigned to four experimental groups: SHAM, SHAM + ischemia, CLP and CLP + ischemia. Each group consisted of 5 hearts. Ischemia was induced by global coronary no-flow at room temperature for 40 min. After 40 min of reperfusion the hearts were weighed and were frozen in liquid nitrogen (Fig. 1a).

### Protocol 2

After a stabilization period of 15 min, hearts were randomly assigned to four experimental groups: SHAM + ischemia, SHAM + AdorAntagonists (SCH58261 + MRS1706) + ischemia, CLP + ischemia, and CLP + AdorAntagonists (SCH58261 + MRS1706) + ischemia. Each group consisted of 10 hearts. Ischemia was induced by global coronary no-flow and kept in a glass cage at constant temperature for 40 min. Antagonists were administered with reperfusion. After 40 min of reperfusion the hearts were weighed and were frozen in liquid nitrogen (Fig. 1b). A selective non-xanthine adenosine AdorA2a antagonist SCH 58261 (*K*_*i*_ = 1.3 nM; Tocris bioscience, Bristol, United Kingdom) and a selective non-xanthine adenosine AdorA2b antagonist MRS1706 (*K*_*i*_ = 1.39 nM; Tocris bioscience, Bristol, United Kingdom) at a concentration of 15 nM were used [20].

### mRNA Extraction and Real-Time PCR Analysis

Total tissue and cell RNAs were extracted from homogenized tissue with TRIzol Reagent (Invitrogen) according to the manufacturer’s instruction. Total RNA was reverse transcribed into cDNA according to standard protocols as described previously [21]. Real-time PCR for AdorA2a, AdorA2b, and β-actin was performed in a LightCycler 480 (Roche, Mannheim, Germany). All PCR experiments were conducted using the LightCycler DNA Master SYBR Green I kit provided by Roche Molecular Biochemicals (Mannheim, Germany) as described previously [22]. The following primers were used: rat AdorA2a (NM_053294) sense: 5′-CCGAATTCCACTCCGGTACA-3′, antisense: 5′-CAGTTGTTCCAGCCCAGCAT-3′; rat AdorA2b (NM_017161) sense: 5′-TCTTCCTCGCCTGCTTCGT-3′, antisense: 5′-CCAGTGACCAAACCTTTATACCTGA-3′; β-actin (NM_031144) sense: 5′-CCGCCCTAGGCACCAGGGTG-3′, antisense: 5′-GGCTGGGGTGTTGAAGGTCTCAAA-3′.

### Protein Preparation and Immunoblotting

Protein preparation and immunoblotting were performed as described previously with some minor modifications [23].

In brief, left ventricles were homogenized and lysed for 15 min in ice-cold lysis buffer (150 mM NaCl, 25 mM Tris (pH8.0), 5 mM EDTA, 1 % Triton X-100) in the presence of a protease inhibitor cocktail (Complete, Roche), followed by centrifugation with 500 g for 15 min at 4 °C. The resultant supernatant was centrifuged at 10,000 g for 30 min at 4 °C. The resultant pellet was reconstituted in blotting buffer and used for Western blotting.

Protein samples (20 μg) from left ventricles were electrophoretically separated on 10 % polyacrylamide gels and transferred to nitrocellulose membranes, which were blocked overnight in 5 % nonfat dry milk diluted in Tris-buffered saline with 0.1 % Tween-20, and then incubated for 1 h at room temperature with antibodies against β-Actin (ab8227, Abcam, Cambridge, United Kingdom; 1:1000), AdorA2a (AB1559, Merck Millipore, Billerica, MA; 1:1000) or AdorA2b (SC28996, Santa Cruz Biotechnology, Santa Cruz, CA; 1:1000). After being washed, the membrane was incubated for 2 h with a horseradish peroxidase–conjugated secondary antibody (Santa Cruz Biotechnology; 1:2000) and subjected to a chemiluminescence detection system. Quantitative assessment of band densities was performed densitometrically using ImageJ Software.

### Statistical Analysis

All data in the text, tables and figures are displayed as means ± standard error of the mean. For statistical analysis, we applied the Kolmogorov- Smirnov test to confirm normal distribution for each group. Raw data from each selected end point were compared by unpaired Student’s *t*-test or by Wilcoxon-Mann–Whitney test, respectively. *P* < 0.05 was considered to be statistically significant. The statistical software used to conduct the analyses was SPSS 16 (SPSS Inc., Chicago, IL, USA).

## Results

**Fig. 2:**
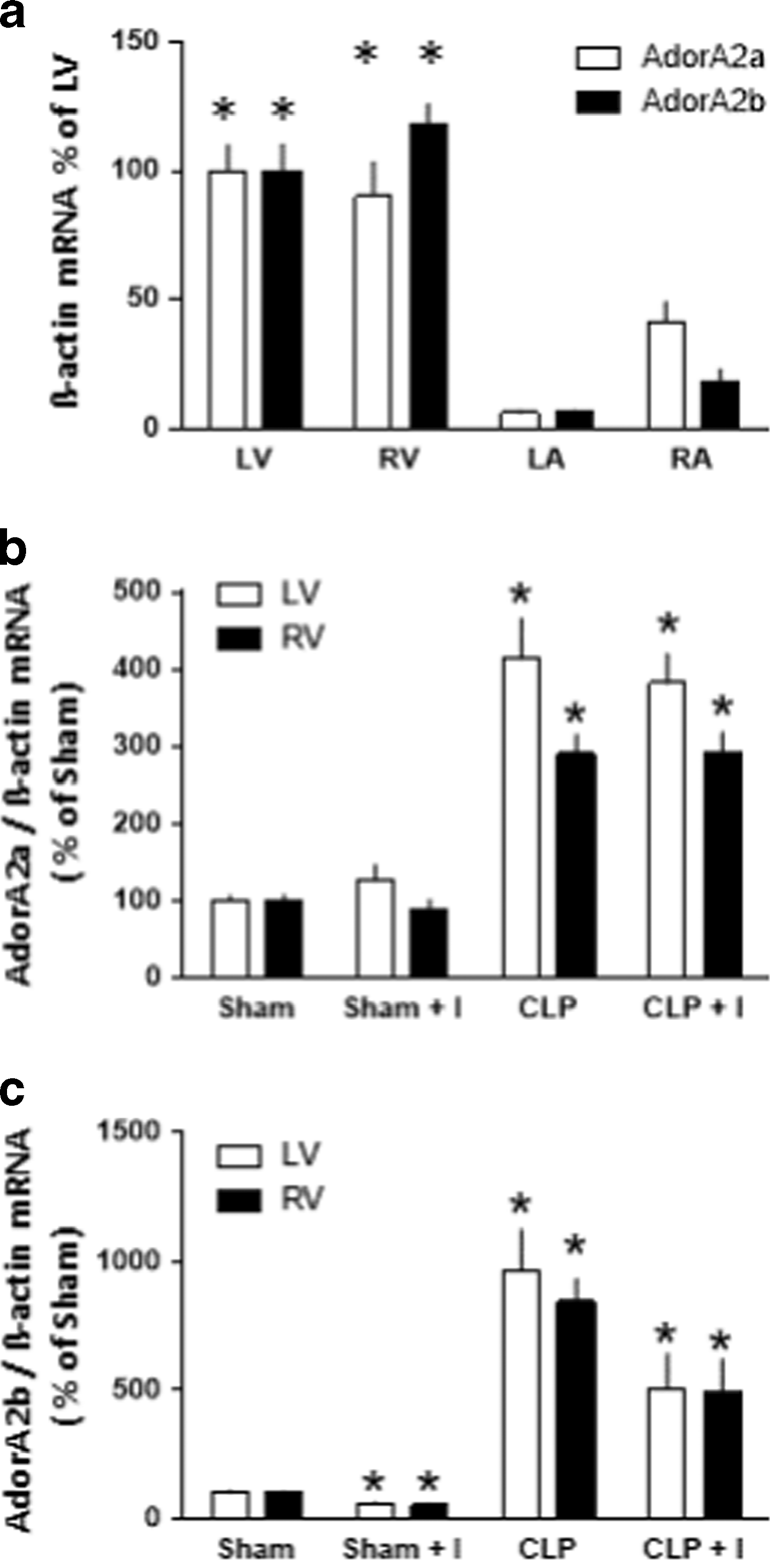
(**a**) AdorA2a and AdorA2b mRNA expression in the left atrium (LA), right atrium (RA), right ventricle (RV), and left ventricle (LV) in SHAM animals. *p < 0.05 ventricle vs. atrium. (**b**) AdorA2a mRNA expression in the right (RV) and left ventricle (LV) in sham- or CLP-treated animal with or without ischemia (I). (**c**) AdorA2b mRNA expression in the right (RV) and left ventricle (LV) in sham- or CLP-treated animal with or without ischemia (I). Data are means ± SEM of 5 animals per group. *p < 0.05 vs. sham-treated animals without ischemia (I)

First, we investigated the distribution of AdorA2a and AdorA2b mRNA in different zones of the rat heart. We found that AdorA2a and AdorA2b mRNA were predominantly expressed in the right and left ventricle (Fig. 2a). Therefore, we determined the effect of CLP on AdorA2a and AdorA2b mRNA expression in the right and left ventricle with or without ischemia (Fig. 2a). CLP for 24 h significantly increased AdorA2a mRNA 4.1- and 2.9-fold in left and right ventricles (*p* < 0.05), respectively. Ischemia and reperfusion for 40 min each did not alter AdorA2a mRNA in SHAM- and CLP-treated animals (Fig. 2b). CLP for 24 h also significantly increased AdorA2b mRNA 9.7- and 8.5-fold in the left and right ventricle, respectively (Fig. 2c). Ischemia and reperfusion for 40 min each decreased AdorA2b mRNA to 57 and 52 % in left and right ventricles of SHAM-treated animals, respectively (Fig. 2a). Furthermore, ischemia attenuated the CLP-induced increase in AdorA2b mRNA in left and right ventricles to about 52 and 58 % of values found for CLP, respectively (Fig. 2c).

**Fig. 3:**
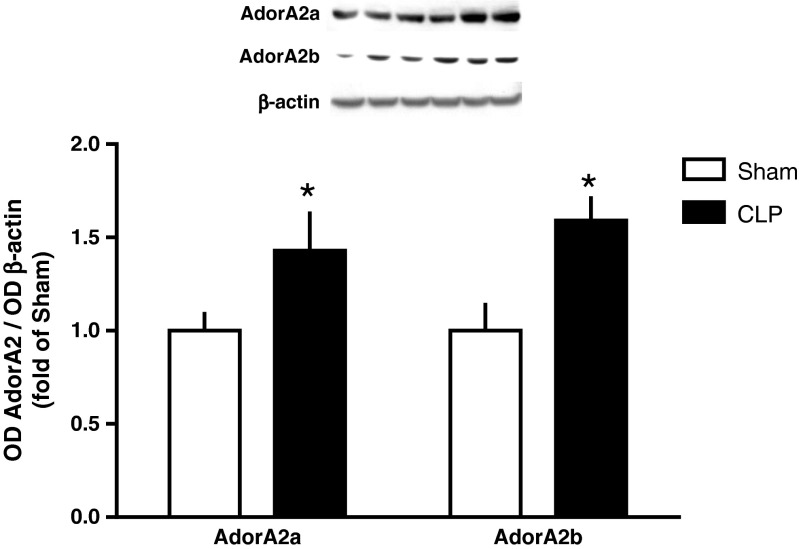
Relative Optical Density (OD) from immunoblots of AdorA2a and AdorA2b of left ventricular tissue 24 h after CLP. Values are given as fold of sham-treated animals. Values are related to signals that were obtained for β-actin. Data are means ± SEM of 3 animals per group. *p < 0.05 vs. sham-treated animals

We further investigated the protein expression of AdorA2a and AdorA2b receptors in left ventricles of rat hearts. We found, that AdorA2a and AdorA2b protein expression significantly increased 1.43-(*p* < 0.05) and 1.59-fold (*p* < 0.05), respectively, in CLP-treated animals compared to SHAM-treated animals (Fig. 3).

**Fig. 4:**
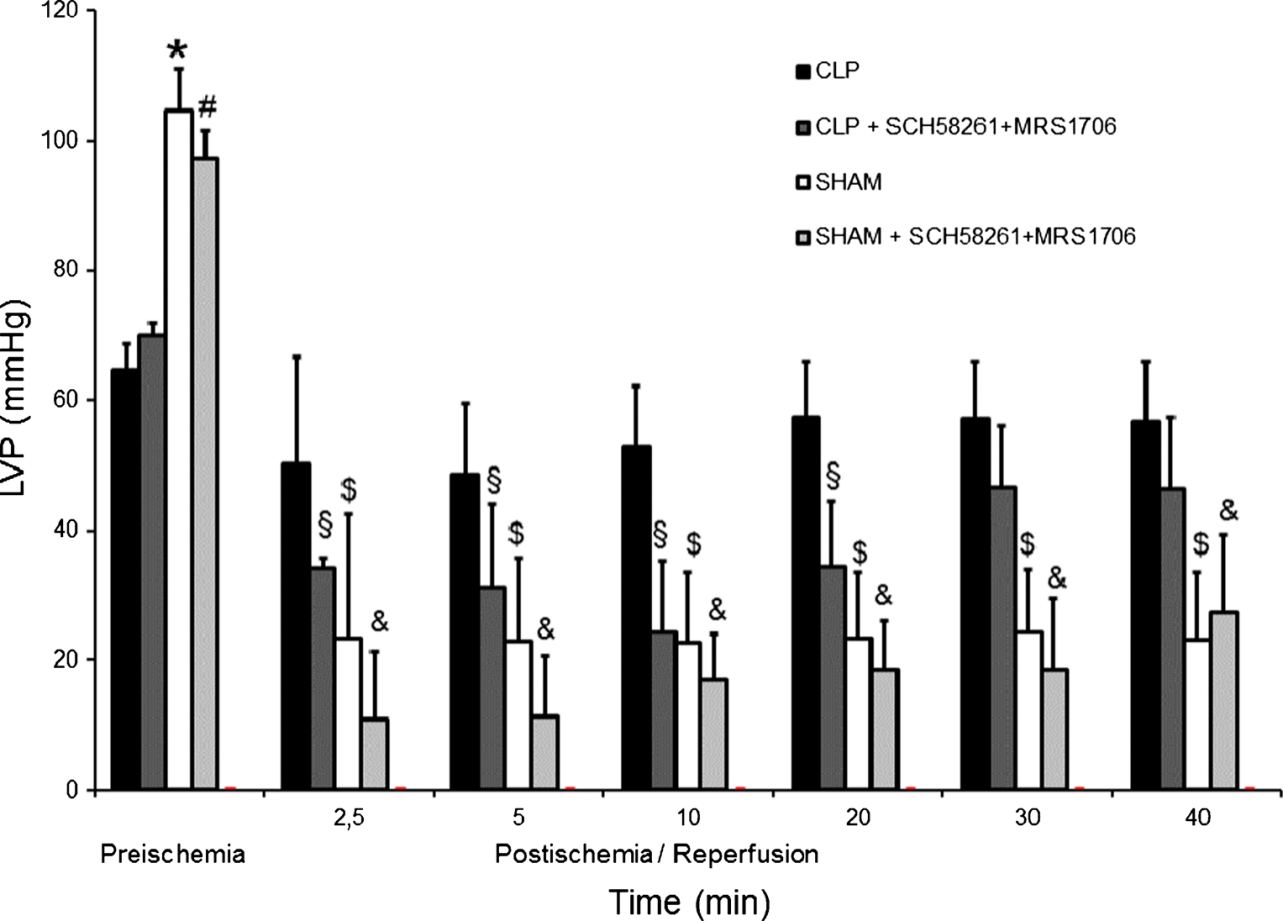
Pre- and post-I/R left ventricular pressure of CLP, CLP+ SCH58261 + MRS1706, SHAM and SHAM+ SCH58261 + MRS1706. Pre-I/R: *p < 0.05 SHAM vs. CLP and CLP + SCH58261 + MRS1706; ^#^ *p* < 0.05 SHAM+ SCH58261 + MRS1706 vs. CLP and CLP + SCH58261 + MRS1706. Post-I/R vs. Pre-I/R: ^§^ *p* < 0.05 for CLP + SCH58261 + MRS1706, ^$^ *p* < 0.05 for SHAM and &*p* < 0.05 for SHAM + SCH58261 + MRS1706. The data are presented as the means ± SEM of 10 animals per group

Baseline values for left ventricular pressure before I/R for SHAM and SHAM + MRS1706 + SCH58261, CLP, and CLP + MRS1706 + SCH58261 were 104 ± 6 mmHg, 97 ± 2 mmHg, 63 ± 5 mmHg and 70 ± 2 mmHg, respectively. Before I/R CLP hearts showed a significant mechanical left ventricular impairment compared to SHAM hearts (Fig. 4). After I/R, a significant LVP reduction was measured in SHAM hearts (24 ± 32 mmHg); CLP hearts LVP remained nearly unchanged (65 ± 13 mmHg) compared to pre-ischemia. Administration of SCH58261 and MRS1706 at reperfusion markedly reduced LVP in the CLP group, and almost diminished the “cardioprotective” effect in septic hearts. In the SHAM + SCH58261 + MRS1706 group, the combined blockade of AdorA2a and AdorA2b led to an electromechanical dissociation (pressureless electrical activity) in 8 of 10 hearts (80 %). An electromechanical dissociation was seen in SHAM and CLP in 40 and 20 %, respectively.

**Fig. 5:**
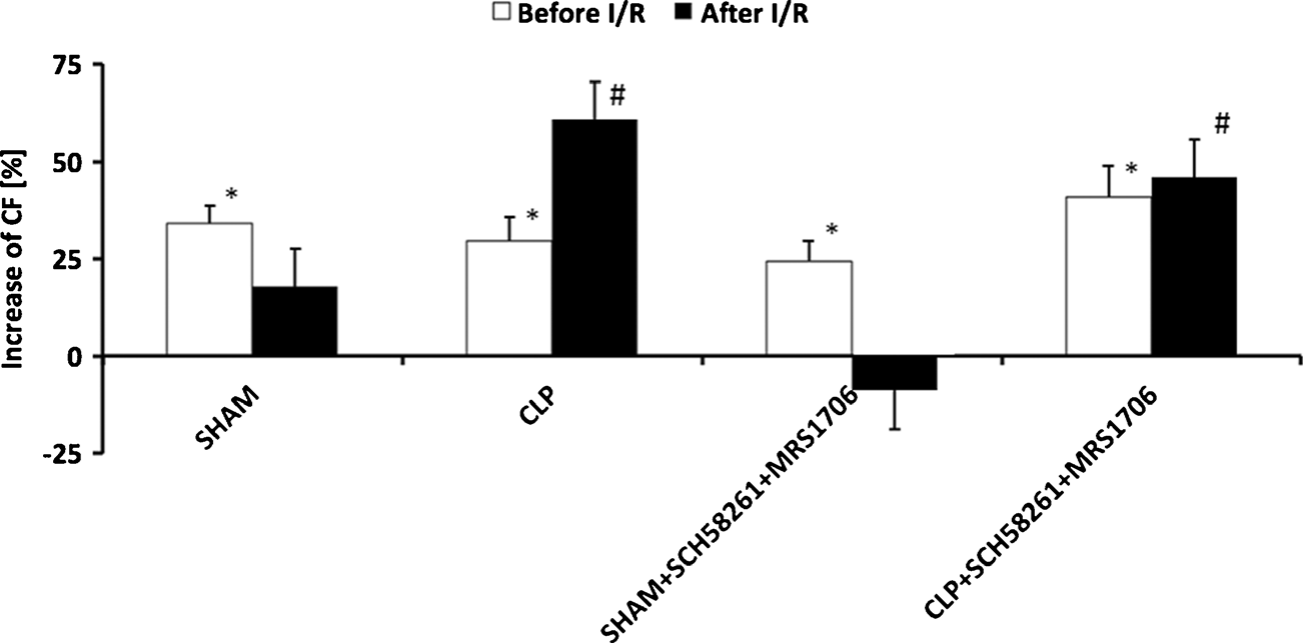
Increase of coronary flow (CF) after administration of adenosine before and after I/R. *p < 0.05 before I/R and ^#^ *p* < 0.05 after I/R for baseline vs. SHAM, SHAM + SCH58261 + MRS1706, CLP, or CLP+ SCH58261 + MRS1706. The data are presented as the means ± SEM of 10 animals per group

**Fig. 6:**
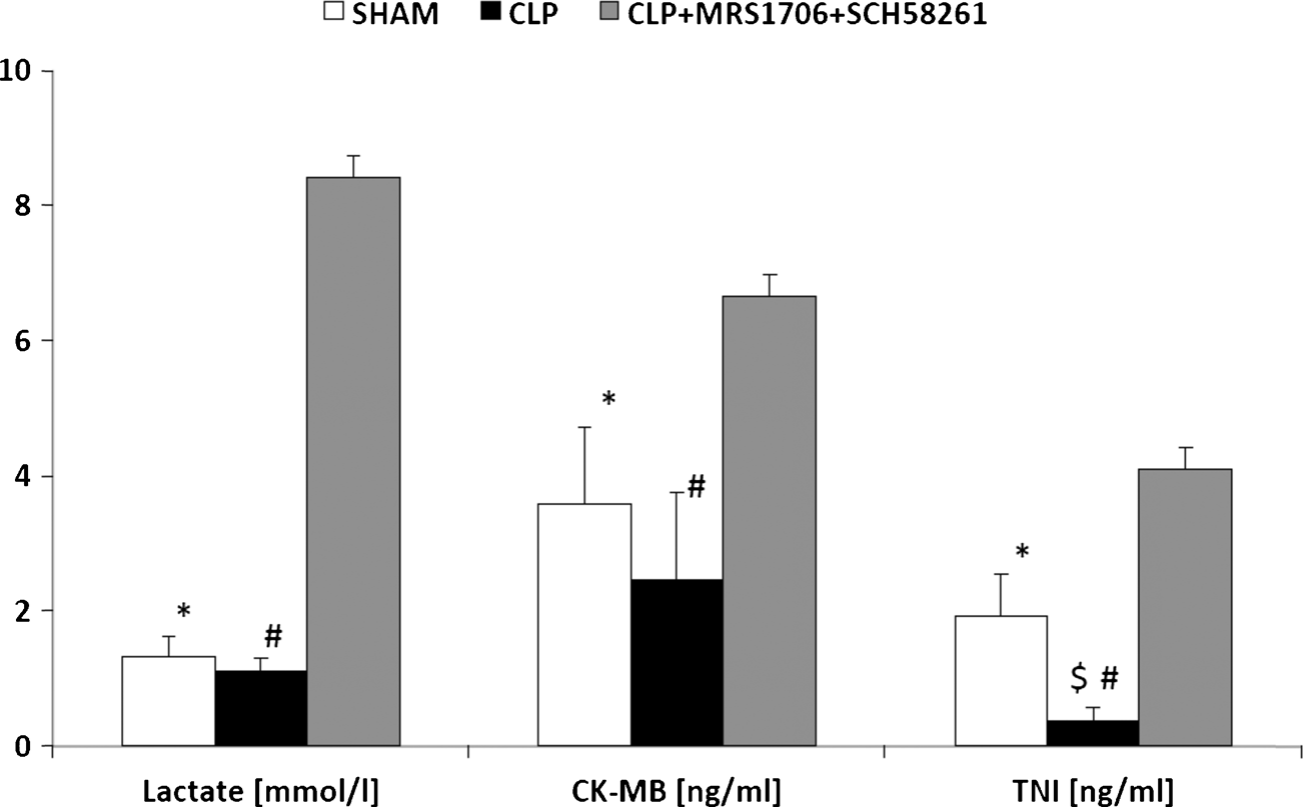
Lactate, CK-MB, and TNI in post-I/R effluat of SHAM, CLP, and CLP + SCH58261 + MRS1706. * and ^#^ *p* < 0.05 SHAM and CLP vs. CLP + SCH58261 + MRS1706, respectively. ^$^ *p* < 0.05 SHAM vs. CLP. The data are presented as the means ± SEM of 10 animals per group

Baseline values for coronary flow before I/R for SHAM, SHAM + SCH58261 + MRS1706, CLP, and CLP + SCH58261 + MRS1706 were 11.5 ± 0.7 ml/min, 11.1 ± 0.4 ml/min, 11.4 ± 0.4 ml/min, 11.2 ± 0.8 ml/min, respectively. Before I/R, maximal coronary flow responses to bolus adenosine administration were significantly increased in all groups compared to baseline values (Fig. 5). After I/R an adenosine reaction was only seen in both CLP groups, not in the other groups. Besides the functional results, we collected cardiac enzymes in the effluent after I/R. Cardiac proteins, like troponin I (TNI), were significantly lower in CLP compared to SHAM. A combined blockade of AdorA2a and AdorA2b in CLP hearts led to significantly increase of lactate, CK-MB, and TNI (*p* < 0.05) (Fig. 6).

## Discussion

The results of the presented study show a reduced morphological and functional damage after ischemia and reperfusion in an isolated septic rat heart model. The maintenance of left ventricular function after a prolonged I/R episode was accompanied by a less pronounced release of cardiac enzyme, troponin I, as indirect measurement of the integrity of the cell. Furthermore, we were able to demonstrate a markedly increase of mRNA and protein expression of AdorA2a and AdorA2b in ventricles of septic hearts. By blocking AdorA2a and AdorA2b at the onset of reperfusion the cardioprotective effect is diminished in septic hearts. Therefore, it seems that Ador plays an important role in the cardioprotection of septic hearts.

In healthy hearts, it is well known that the activation of adenosine receptors play a major role for cardioprotection [9–11]. However, little is known about this mechanism of I/R injury in non-healthy hearts, especially in septic hearts. This is of prominent interest as sepsis goes along with a variety of a variable hemodynamic with long and lasting episodes of hypotension, a dysfunction of the microvascular bed and the administration of vasoactive drugs [2, 3]. All of these may compromise the coronary integrity that may lead to ischemia and exposing the septic hearts to increased risk.

However, it seems that in sepsis hearts are cardioprotected. McDonough et al. were one of the first showing a reduced I/R injury, after a short and even a long term ischemic episode in septic hearts [6, 8]. After ischemia septic hearts show mechanical and morphological reperfusion injury to a lesser extent than sham hearts [6, 7]. These results concur with ours. After I/R left ventricular function was reduced is SHAM hearts, but not in CLP hearts. Adding Ador-Antagonists at reperfusion abolished the cardioprotection of CLP hearts.

There is growing evidence that adenosine receptor may play a major role for cardioprotection in cardiovascular disease, eg in diabetes [24]. For example, A_3_ receptor might be a possible cardioprotectant in diabetes, which is currently under extensive investigation [24]. Therefore we investigated if adenosine receptors may play a role for the cardioprotection seen in septic hearts. We showed an increased expression of mRNA and protein for AdorA2a and AdorA2b in the septic hearts. A combined blockade of these receptors led to a significant decrease in the left ventricular function after I/R in these hearts. This could be interpreted that the activation of both receptors (AdorA2a and AdorA2b) are necessary for the cardioprotection of the septic heart. This hypothesis is supported by a recent study from Xi and colleagues [20]. They reported that AdorA2a and AdorA2b need to work in concert to produce a strong and consistent protection against reperfusion injury in rat hearts. Furthermore, Zhan et al. showed recently, that both AdorA2a and AdorA2b are required for adenosine A_1_ receptor-mediated cardioprotection, which implicated an interaction among the receptor subtypes [12]. Recently, McIntosh and Lasley pointed out that for the cardioprotection there is significant evidence that all 4 subtypes are important [11].

Studies in healthy animals have shown that the activation of AdorA2a prior to ischemia does not result in a reduction of myocardial I/R injury [11]. However, stimulation of AdorA2a at the onset of reperfusion leads to markedly decrease in I/R injury. For example, Norton et al., showed a dose dependent effect of a selective AdorA2a agonist CGS-21680 leading to a significant reduction in histologically determined infarct size [13]. In addition, other studies in a similar model showed, that the application of antagonists (SCH58261 or MRS1706) at reperfusion reverses the infarct size reducing effects of AdorA2 agonists [20]. We administered the antagonists at reperfusion, which led to a decreased left ventricular function and a pronounced release of cardiac enzyme as indirect measurement of cellular destruction in the “once protected” septic heart. This assumes that “cardioprotection” in septic hearts is probably generated at the phase of reperfusion. However, other pathways or mechanism (eg MAPK-dependent mechanism) might also be important for the “cardioprotection” in septic hearts [25].

Clinical studies of septic patients showed an alternated coronary function with a high coronary blood flow and a diminished coronary artery-coronary sinus oxygen difference [26]. Based on these findings and a generalized vascular dysfunction in mind, a disturbed coronary flow autoregulation has been speculated [3]. Our Langendorff apparatus, is a well recognized model that allows examination of direct coronary vascular effects independent of autoregulation [27, 28]. Therefore, as sepsis-induced alteration of coronary flow before I/R can be ruled out in this model. Furthermore, only CLP hearts exhibited sufficient coronary reserve after I/R, which indicates that coronary autoregulatory capability was only intact in CLP and not in the other groups. Consequently, the up-regulation of adenosine receptors may have led to the maintenance of coronary autoregulation in CLP, and I/R may have triggered the excessive effect. This may be the mechanism behind the cardioprotection seen in septic hearts. Furthermore, based on other studies metabolic effects, like level of high energy phosphate, amount of anaerobic glycolysis or myocardial catalase levels are less likely to be the reason for cardioprotection [6, 8].

We do understand well that this study has limitations. Although the applied sepsis model has several advantages (e.g. ‘natural’ course of infection, closely mimicing human disease by activating pro- and anti-inflammatory pathways), there are some limitations, especially with regard to noteworthy outcome variability [29, 30]. In contrast, bolus injection-sepsis models indeed offer a simple and highly standardized method, but it has been shown that they do not reflect all aspects of the sepsis syndrome [29, 30]. However, as other studies using a different sepsis model showed comparable results, the model per se might not have been a limitation [6, 8].

Furthermore, we did not apply different Ador-antagonists. We concentrated on SCH58261 and MRS1706. We chose these antagonists as they are more selective for AdorA2a (100-323-fold) and AdorA2b (113-165-fold) than for AdorA1 or AdorA3, respectively [20]. However, we can not rule out that an activation or blockade of AdorA1 and/or AdorA3 might have influenced our results and conclusion. Furthermore, other possible causes for the cardioprotection in sepsis, eg up-regulation of iNOS were not part of our investigation, and their relevance might be underestimated [3].

### Conclusion

The morphological and functional I/R injury in septic animals is less pronounced compared to healthy hearts. By application of combined AdorA2a + b antagonists this “cardioprotective” effect is reduced in septic hearts. Our results suggest for the first time that septic hearts are more or less cardioprotected, the adenosine receptors AdorA2a and AdorA2b play predominant role for a reduced morphological and functional I/R injury in the septic heart. It is plausible that an intact coronary autoregulation play the most important role for the cardioprotection seen in septic hearts.

## References

[1] Singer M, Deutschman CS, Seymour CW, Shankar-Hari M, Annane D, Bauer M (2016). The third international consensus definitions for sepsis and septic shock (sepsis-3). JAMA.

[2] Dellinger RP, Levy MM, Rhodes A, Annane D, Gerlach H, Opal SM (2013). Surviving sepsis campaign: International guidelines for management of severe sepsis and septic shock, 2012. Intensive Care Med.

[3] Merx MW, Weber C (2007). Sepsis and the heart. Circulation.

[4] Hinshaw LB (1996). Sepsis/septic shock: participation of the microcirculation: an abbreviated review. Crit Care Med.

[5] Westerhof N, Boer C, Lamberts RR, Sipkema P (2006). Cross-talk between cardiac muscle and coronary vasculature. Physiol Rev.

[6] McDonough KH, Causey KM (1994). Effects of sepsis on recovery of the heart from 50 min ischemia. Shock.

[7] McDonough KH, Lang CH, Spitzer JJ (1984). Depressed function of isolated hearts from hyperdynamic septic rats. Circ Shock.

[8] McDonough KH, Causey KM (1994). Sepsis protects the heart of alcoholic rats from ischemia-reperfusion injury. Alcohol Clin Exp Res.

[9] Chen JF, Eltzschig HK, Fredholm BB (2013). Adenosine receptors as drug targets--what are the challenges?. Nat Rev Drug Discov.

[10] Eltzschig HK (2013). Extracellular adenosine signaling in molecular medicine. J Mol Med.

[11] McIntosh VJ, Lasley RD (2012). Adenosine receptor-mediated cardioprotection: Are all 4 subtypes required or redundant?. J Cardiovasc Pharmacol Ther.

[12] Zhan E, McIntosh VJ, Lasley RD (2011). Adenosine a(2)a and a(2)b receptors are both required for adenosine a(1) receptor-mediated cardioprotection. Am J Physiol Heart Circ Physiol.

[13] Norton ED, Jackson EK, Turner MB, Virmani R, Forman MB (1992). The effects of intravenous infusions of selective adenosine a1-receptor and a2-receptor agonists on myocardial reperfusion injury. Am Heart J.

[14] Thornton JD, Liu GS, Olsson RA, Downey JM (1992). Intravenous pretreatment with a1-selective adenosine analogues protects the heart against infarction. Circulation.

[15] Auchampach JA, Jin X, Moore J, Wan TC, Kreckler LM, Ge ZD (2004). Comparison of three different a1 adenosine receptor antagonists on infarct size and multiple cycle ischemic preconditioning in anesthetized dogs. J Pharmacol Exp Ther.

[16] Philipp S, Yang XM, Cui L, Davis AM, Downey JM, Cohen MV (2006). Postconditioning protects rabbit hearts through a protein kinase c-adenosine a2b receptor cascade. Cardiovasc Res.

[17] Ge ZD, van der Hoeven D, Maas JE, Wan TC, Auchampach JA (2010). A(3) adenosine receptor activation during reperfusion reduces infarct size through actions on bone marrow-derived cells. J Mol Cell Cardiol.

[18] Zausig YA, Busse H, Lunz D, Sinner B, Zink W, Graf BM (2009). Cardiac effects of induction agents in the septic rat heart. Crit Care.

[19] Zink W, Kaess M, Hofer S, Plachky J, Zausig YA, Sinner B (2008). Alterations in intracellular ca2 + −homeostasis of skeletal muscle fibers during sepsis. Crit Care Med.

[20] Xi J, McIntosh R, Shen X, Lee S, Chanoit G, Criswell H (2009). Adenosine a2a and a2b receptors work in concert to induce a strong protection against reperfusion injury in rat hearts. J Mol Cell Cardiol.

[21] Hocherl K, Schmidt C, Kurt B, Bucher M (2008). Activation of the pgi(2)/ip system contributes to the development of circulatory failure in a rat model of endotoxic shock. Hypertension.

[22] Hocherl K, Schmidt C, Kurt B, Bucher M (2010). Inhibition of nf-kappab ameliorates sepsis-induced downregulation of aquaporin-2/v2 receptor expression and acute renal failure in vivo. Am J Physiol Renal physiol.

[23] Grenz A, Osswald H, Eckle T, Yang D, Zhang H, Tran ZV (2008). The reno-vascular a2b adenosine receptor protects the kidney from ischemia. PLoS Med.

[24] Nishat S, Shabir H, Azmi AS, Ansari HR (2012). A(3) adenosine receptor: a plausible therapeutic target for cardio-protection in diabetes. Recent Pat Cardiovasc Drug Discov.

[25] Walshe CM, Laffey JG, Kevin L, O’Toole D (2015). Sepsis protects the myocardium and other organs from subsequent ischaemic/reperfusion injury via a mapk-dependent mechanism. Intensive Care Med Exp.

[26] Cunnion RE, Schaer GL, Parker MM, Natanson C, Parrillo JE (1986). The coronary circulation in human septic shock. Circulation.

[27] Dole WP (1987). Autoregulation of the coronary circulation. Prog Cardiovasc Dis.

[28] Zausig YA, Stowe DF, Zink W, Grube C, Martin E, Graf BM (2006). A comparison of three phosphodiesterase type iii inhibitors on mechanical and metabolic function in guinea pig isolated hearts. Anesth Analg.

[29] Dejager L, Pinheiro I, Dejonckheere E, Libert C (2011). Cecal ligation and puncture: the gold standard model for polymicrobial sepsis?. Trends Microbiol.

[30] Hubbard WJ, Choudhry M, Schwacha MG, Kerby JD, Rue LW, Bland KI (2005). Cecal ligation and puncture. Shock.

